# Exploring the impact of Patient Reported Outcome Measures (PROMs) among orthopaedic surgeons in mainland China: systematic review and survey-based study on hip and knee instruments

**DOI:** 10.1186/s12891-021-04459-3

**Published:** 2021-06-21

**Authors:** James Reeves Mbori Ngwayi, Jie Tan, Ning Liang, Kenedy Uzoma Obie, Daniel Edward Porter

**Affiliations:** 1 School of Clinical Medicine, Tsinghua University, Zijing Apartment 21, Beijing, 100084 China; 2 School of Clinical Medicine, Central South University, Changsha, 410011 China; 3 Department of Orthopaedics, First (Huaxin) Hospital of Tsinghua University, Beijing, 100016 China

**Keywords:** Hip PROMs, Knee PROMs, Systematic literature review, China

## Abstract

**Background:**

Patient Reported Outcome Measures (PROMs) are widely used in Europe and North America in a variety of areas including research, clinical governance, clinical registries and insurance ascertainment. The aim of this study was to assess commonly used knee and hip PROMs among Chinese surgeons and to gain an insight into their impact on evaluation of clinical outcomes.

**Methods:**

1. A systematic literature search of databases Medline, EMBASE, CINAHL and CNKI was performed from the earliest records to 22/07/2020 for knee instruments and 22/08/2020 for hip instruments, to retrieve Chinese Mandarin cross culturally adapted and validated knee and hip PROMs. 2. An 11-item electronic questionnaire was then designed under four domain categories. The survey was distributed via a ubiquitous online social media platform to orthopaedic surgeons. Responses were collected and analyzed. Output from 1. was used to populate parts of the survey questionnaire.

**Results:**

The systematic online search yielded a total of 41 evaluation instruments, (10 hip and 31 knee); all of which were incorporated as response options. 234 viable questionnaires were retrieved with the largest group representing attending surgeons. 59.0% were familiar with the concept of PROMs among which 78.4% reported to have used PROMs themselves. In order of frequency of use, PROMs were purposed for clinical assessment (55.6%), research (40.7%), health regulation policies (18.6%) and insurance service requirements (10.6%). Implementation was prompted by both departmental (43.4%) and institutional policy (34.5%). 89.4% of PROMs users reported difficulties in the use of PROMs, with major barriers including license fees, limited access, inadequate training and burden of fill-out time (all > 40%).

**Conclusion:**

There is evidence of limited familiarity with knee and hip PROMs among orthopaedic surgeons. Barriers to their use are significant. Development of a Chinese language PROMs database would be helpful.

**Supplementary Information:**

The online version contains supplementary material available at 10.1186/s12891-021-04459-3.

## Introduction

PROMs are subjective self-reported questionnaires, typically providing information on individual patients’ health status directly without clinician interpretation [1]. The revolution in evidence-based medicine has placed the patient at the center of clinical decision-making, promoting a doctrine of the medical service which is geared at improving patient symptoms, function and quality of life [2]. The advantages of PROMs and their applicability in comparing outcomes from different health providers, identifying strengths and deficiencies in health care delivery, boosting quality improvement, and promoting choice are increasingly evident [3].

Orthopedic arthroplasty registries have recognized the importance of PROMs and have incorporated general and specific PROMs such as the Oxford Hip Score (OHS), WOMAC (The Western Ontario and McMaster Universities Osteoarthritis Index) and 12-item Short Form Survey (SF-12) as part of patient function and quality of life assessment [4].

In the UK, where the use of certain locomotor PROMs is mandated by commissioning bodies, for example within the referral pathway for knee replacement [5], anecdotal evidence suggests PROMs use in primary care is fragmented and unsystematic; suggesting that studies to explore attitudes of clinicians towards PROMs use could be helpful [6]. In China, development of the primary care system is embryonic and does not have a ‘gate-keeping’ function [7]. The Chinese government is expanding the role of primary care for patients with common degenerative conditions. It is therefore to be hoped that general practitioners could become familiar with suitable Chinese-language validated PROMs in the future. However, their current impact is mainly in the secondary care sector. Although PROMs have been developed for use in Traditional Chinese Medicine [8], their utility in surgery may not be well understood since there is no literature exploring surgeon comprehension or use of PROMs in clinical practice or research in China. In 2019 it was estimated that nearly one million joint replacements were carried out in China with an annual growth rate of 38% [9].. The aim of this survey was to 1. explore knowledge of and attitudes towards commonly used knee and hip PROMs among Chinese orthopedic surgeons, 2. identify barriers to PROMs use, and 3. gain an insight into their impact on evaluation of clinical outcomes.

## Methods

### Identification of Chinese cross culturally adapted and original Chinese knee and hip PROMs

We performed separate online database searches for Hip and Knee assessment tools on the following databases PubMed/MEDLINE, EMBASE (OVID), CINAHL (EBSCO) and CNKI (mainland Chinese index database) with methodology compliant with previous recommendations [10]. Retrieved reports dated from earliest records till end July 2020. Sensitive filters were designed using MeSH terms and keyword combinations based on previously described strategies for PROMs searches [11–13] (Additional file 1 filter); with inclusion criteria as follows: 1. Cross culturally adapted and original Chinese hip and knee PROMs. 2. Tested on the mainland Chinese population. 3. Chinese Mandarin as language of cross-cultural adaptation.

Screening was carried out as a 3-step process (Titles, Abstract and Full texts), each performed by two orthopedic surgeons fluent in the language. Following independent review consensus was reached on articles to be retrieved. After full-text screen and identification of suitable articles, references were manually reviewed to identify missing articles which were not part of the electronic search output.

### Questionnaire design

We designed an 11-item questionnaire in 4 domains: Surgeon details (questions 1 and 2); Surgeon knowledge (questions 3–5); PROMs usage (questions 6–9); PROMs barriers (questions 10 and 11). Questions were modified based on adaptations from previous similar studies [14]. The following items were included in the questionnaire:

1. Are you an Orthopedic Surgeon?
2. What level of training are you?
3. Do you know about PROMs?
4. Which Knee Scores are you familiar with?
5. Which Hip Scores are you familiar with?
6. Have you used Knee or Hip PROMs before?
7. Which Hip Scores have you used before?
8. Which Knee Scores have you used before?
9. For what purpose do/did you use PROMs?
10. Do you have any difficulties using PROMs?
11. What difficulties hinder you from using PROMs?

Response options were of mixed format, including binary, multiple choice and free-text options. Results of the systematic search for knee and hip tools were incorporated as response options for questions 4 and 5 respectively, with a capacity for respondents to add other PROMs not on the list. Answers to questions 7 and 8 were open-ended free-text. To increase accuracy of responses, the questions were encrypted with a logic coding whereby a negative answer to question 1,3,6 and 10 prevented display of further questions, automatically ending the questionnaire.

The questionnaire was designed using the online platform WenJuanXing® (Changsha RanXing Information Technology Co., Ltd), and was compatible for smart gadgets. Distribution of the electronic questionnaire was done online via the ubiquitous social media platform the WeChat®, targeting different orthopedic groups. The survey was sent to orthopedic surgeons over a 4-week period from 24 September 2020 to 24 October 2020. Sampling was purposive and intended to capture orthopaedic surgeons of all grades (residents, attending surgeons, vice directors and directors). An online network of Chinese Orthopedic Association conference attendees (2019), a provincial city-level orthopedic association group and university health-system affiliated hospital orthopedic department groups were engaged. Onward dissemination of the survey among fellow orthopedic colleagues was encouraged. Responses were automatically collected and indexed on the same online platform. Analysis and graphics were designed with Microsoft Excel® 2018 (Microsoft, Redmond, WA, USA). Statistical display and analysis was descriptive.

## Results

**Fig. 1:**
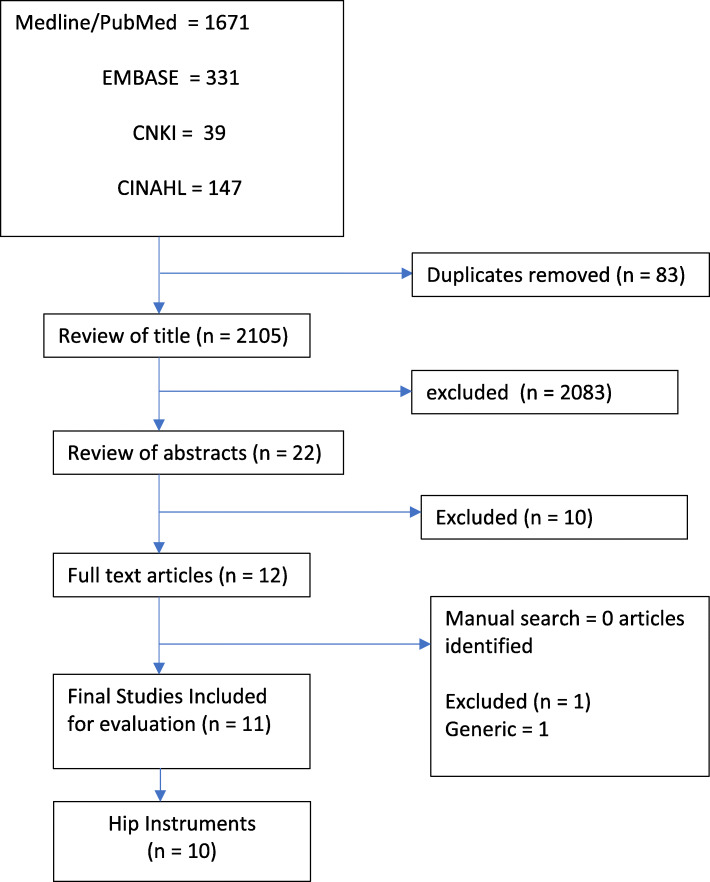
PRISMA FLOW CHART – HIP instruments

**Fig. 2:**
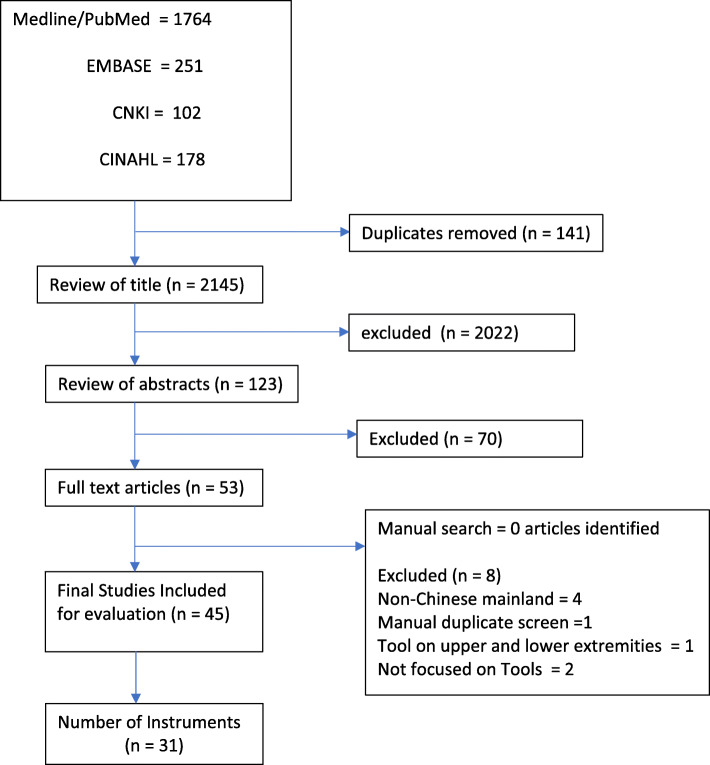
PRISMA FLOW CHART – Knee instruments

The systematic online search yielded a total of 41 instruments, (10 hip and 31 knee). The PRISMA chart of the review process is illustrated in Figs. 1 and 2. All of these were included in the surgeon questionnaire as multiple-choice options (Additional file 2 Table, Additional file 3 table references).

A total of 305 responses were retrieved and 234 were viable following exclusion of 71 respondents who were not orthopedic surgeons. Current training level was Resident 18.4%, Attending Surgeon 45.7%, Vice Director 29.1% and Director 6.8%.

**Fig. 3:**
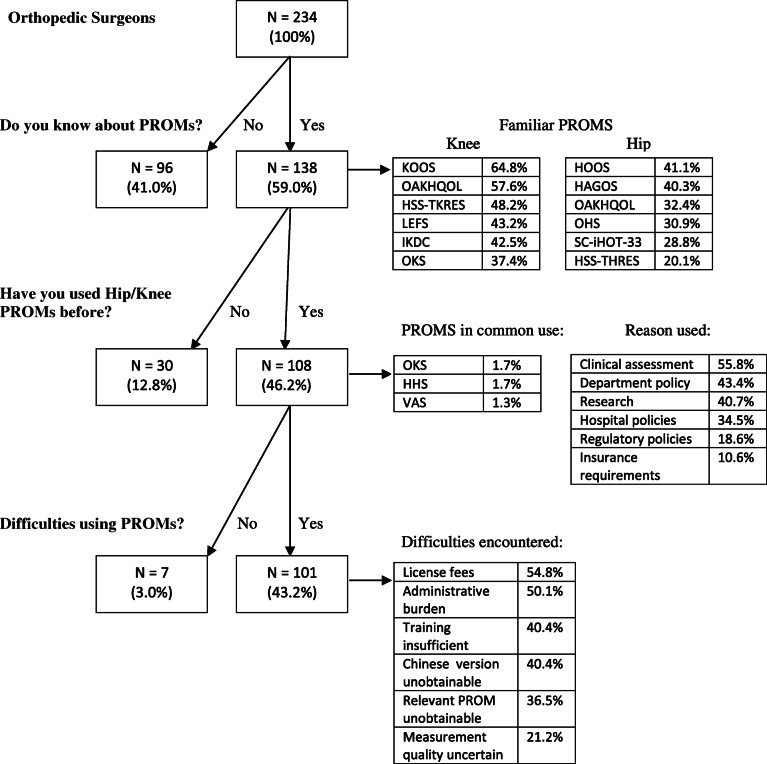
Flow chart of Responses. Key: KOOS:Knee injury and Osteoarthritis Outcome Score; OAKHQOL: Osteoarthritis of Knee and Hip Quality of Life; HSS-TKRES: Hospital for special surgery total knee replacement expectations survey; LEFS: Lower Extremity Function Scale; IKDC: International Knee Documentation Committee; OKS: Oxford Knee Score; HOOS: Hip disability and Osteoarthritis Outcome; HAGOS: Copenhagen Hip and Groin Outcome Score; OHS: Oxford Hip Score; SC-iHOT-33: International Hip Outcome Tool; OKS:Oxford Knee Score; HHS: Harris Hip Score; VAS: Visual Analogue Scale

The flowchart of responses is shown in Fig. 3 together with the list of PROMs which were identified in response to questions 4,5,7 and 8. The reasons for using PROMs and barriers to their introduction are also documented. 59.0% of respondents were familiar with the concept of PROMs, and the most familiar knee PROMs were: The Knee injury and Osteoarthritis Outcome Score (KOOS), International Knee Documentation Committee (IKDC), Oxford Knee Score (OKS),The Osteoarthritis of Knee and Hip Quality of Life (OAKHQOL), Hospital for Special Surgery Total Knee Replacement Expectations Survey (HSS-TKRES), Lower Extremity Function Scale (LEFS). Among those who knew about PROMs, the above six PROMs achieved recognition rates of over 35% (Fig. 3). Most familiar hip PROMs were: Hip disability and Osteoarthritis Outcome (HOOS), Oxford Hip Score (OHS), Copenhagen Hip and Groin Outcome Score (HAGOS), International Hip Outcome Tool (SC-iHOT-33), Osteoarthritis of Knee and Hip Quality of Life (OAKHQOL), Hospital for Special Surgery Hip Replacement Expectations Survey (HSS-THRES). Among those who knew about PROMs, the above six PROMs achieved recognition rates over 20% (Fig. 3). Among all surgeons, 46.2% reported to have used PROMs themselves. Named PROMs that had been used included Oxford Knee Score (OKS) and the Harris Hip Score (HHS). Surgeons use PROMs primarily for clinical assessment and research, as mandated by either departmental or hospital policy (all > 30%). Major difficulties using PROMs were high license fees, patient time burden, insufficient training and difficulties obtaining Chinese language versions (all > 40%).

## Discussion

The majority of Orthopedic surgeons in smaller cities in Mainland China work in general orthopedic wards addressing a wide variety of musculoskeletal disorders. Most doctors work at attending surgeon level, so respondent seniority profile is likely to be representative of Chinese orthopedic surgeons overall. Most surgeons (59.0%) were familiar with the concept of PROMs. The Knee injury and Osteoarthritis Outcome Score (KOOS) and the Hip disability and Osteoarthritis Outcome (HOOS) were the most familiar in each group. Hip PROMs were generally less well known than Knee PROMs. This might reflect the predominance of knee over hip osteoarthritis in east Asian populations [15]. Although 46.2% of respondents reported use of PROMs before, very few were able to name those they had used. Possible reasons for lack of engagement with this question is that previous use of PROMs may have been unstructured and spasmodic. The VAS score is very commonly used as a pain assessment tool in orthopedic units; a similar study on common foot and ankle PROMs also reported that VAS was most commonly used [14]. We had previously performed an online literature search on CNKI and Wangfangdata (Mainland China databases) to identify publications using knee PROMs for 2018 (unreported data). Results identified the Lysholm, VAS and the International Knee Documentation Committee (IKDC) scores as most commonly occurring in literature. The IKDC was also amongst the six most familiar knee articles in this survey.

The commonest response as to why surgeons choose to use PROMs was for clinical assessment and for research, however the difficulties encountered in describing PROMs used suggests that deep familiarity and frequent usage is unlikely. Responses suggest institutional encouragement for the use of PROMs, although their use in routine follow-up is uncertain. Responses as a whole indicate very limited national or provincial policy mandating the use of PROMs (this option checked by10.6% of respondents).

Arthroplasty Registries have existed since 1975 and have adopted PROMs as part of endpoint assessment highlighting patient quality of life and functionality [4]. In several countries PROMs are incorporated into actual clinical practice with central funding of online databases such as FORCE-TJR for collection interpretation and analysis of data in the orthopedic community [16]. No schemes exist yet in Mainland China as reflected in the current study’s surgeon responses.

The majority of PROMs users reported difficulties (89.4%). We did not allow respondents who did not use PROMs to answer this question, so it is possible that these individuals had also experienced difficulties which prevented their use at all. Prominent issues include: high license fees, difficulty finding Chinese versions of the PROMs, and long time spent administering PROMs. Although most PROMs are free-to-use others require fees; this may force clinicians to find alternative instruments. Most PROMs are originally designed in English and thus need to be rigorously translated before implementation in other language settings. Many translated versions are found on English language websites owned by the originator, adding to the difficulties of access and comprehension. Although a wide variety of tools have been cross culturally adapted and validated in mainland Chinese populations, some articles have excluded the Chinese language PROM from publication due to license fee requirements. This may have encouraged the development of multiple translated versions of original PROMs [17]. Surgeons reported that time constraints in completing PROMs was one of the main difficulties. A previous study on general practitioners’ views on the use of PROMs also found this was the main barrier [6]. Thus, the administrative burden is an important aspect that has to be considered to facilitate their clinical use.

Some linguistic regions have developed online PROMs libraries, for example in Spain via BibliPRo (https://www.bibliopro.org) which provides surgeons with search options and download portals. These ensure quality control via assessment of available PROMs using tools such as the Evaluation Measurement of Patient Reported Outcomes (EMPRO) and Consensus Based Standards for the Selection of Health Measurement Instruments (COSMIN) [18–20]. Similar initiatives should be undertaken in Mainland China to ascertain and assemble high quality PROMs for use by future arthroplasty registries, for research and in clinical management. Studies on modalities of PROMs collection will be useful to provide evidence on efficient time saving methods to improve PROMs returns and reduce patient and administrative burden through the advent of electronic PROMs delivered online. Surgeon and patient education will be vital to improve compliance and understanding of PROMs, setting the stage for wider clinical use.

## Limitations

We used purposive and snowball sampling so as to ascertain orthopaedic surgeons in different geographical settings and of differing seniority. However this valid sociological method to sample hard-to-reach groups is a non-random method and has inherent selection biases which are uncontrolled. The results of our survey on attitude may therefore not represent all views or even representative views of orthopaedic surgeons although we have no reason to believe they do not.

## Conclusion

A wide variety of lower limb arthroplasty tools have been translated into Chinese, with the most familiar hip and knee scores being the Hip Disability and Osteoarthritis Outcome (HOOS) and the Knee injury and Osteoarthritis Outcome Score (KOOS). Use of PROMs is generally for research and clinical assessment. Most surgeons reported difficulties in accessing PROMs. Improvements in PROMs utility may rely on a Chinese language database to allow better access and quality control of available PROMs.

## Supplementary Information

**Additional file 1.** Hip and Knee search Filters. PubMed/MEDLINE filter with mesh terms and keywords to search for scores adapted for the Chinese population.

**Additional file 2.** Available Chinese adapted Instruments for Knee and Hip assessment. List of instrumeents and year published for the available Chinese adapted and designed Knee and Hip scores.

**Additional file 3.** Chinese knee and hip score references. List of references for all the scores included in table.

## Data Availability

The datasets used and/or analyzed during the current study are available from the corresponding author on reasonable request.

## Abbreviations

PROMs: Patient Reported Outcome Measures
OHS: Oxford Hip Score
WOMAC: The Western Ontario and McMaster Universities Osteoarthritis Index
SF-12: 12-item Short Form Survey
KOOS: The Knee injury and Osteoarthritis Outcome Score
IKDC: International Knee Documentation Committee
OKS: Oxford Knee Score
OAKHQOL: The Osteoarthritis of Knee and Hip Quality of Life
HSS-TKRES: Hospital for Special Surgery Total Knee Replacement Expectations Survey
LEFS: Lower Extremity Function Scale
HOOS: Hip disability and Osteoarthritis Outcome
HAGOS: Copenhagen Hip and Groin Outcome Score
SC-iHOT-33: International Hip Outcome Tool
OAKHQOL: Osteoarthritis of Knee and Hip Quality of Life
HSS-THRES: Hospital for Special Surgery Hip Replacement Expectations Survey
EMPRO: Evaluation Measurement of Patient Reported Outcomes
COSMIN: Consensus Based Standards for the Selection of Health Measurement Instruments
