# Supplementary material for: Exploring the impact of Patient Reported Outcome Measures (PROMs) among orthopaedic surgeons in mainland China: systematic review and survey-based study on hip and knee instruments
Source: BMC Musculoskelet Disord. 2021 Jun 21;22:566. doi: 10.1186/s12891-021-04459-3 (PMC8218500; doi:10.1186/s12891-021-04459-3)
Supplement: Supplementary file 2 — Additional file 2. Available Chinese adapted Instruments for Knee and Hip assessment. List of instrumeents and year published for the available Chinese adapted and designed Knee and Hip scores. [file 12891_2021_4459_MOESM2_ESM.docx]

**TABLE**

**Table 1 – Available Chinese adapted Instruments for Knee and Hip assessment**

| **Full Name** | **First Published Year/date** |
| --- | --- |
| **Knee Tools (n=31)** |  |
| 1. International Knee Documentation Committee(IKDC) | Zhen-Yu Jia et al (2018) |
| 2. The Knee injury and Osteoarthritis Outcome Score(KOOS) | Wang Jian Sheng（2011） |
| 3. LYSHOLM Score | Wang et al(2016) |
| 4. Oxford Knee Score(OKS) | Lin Kai et al (2017) |
| 5. Western Ontario and McMaster universities Osteoarthritis Index(WOMAC) | Tara Symonds et al (2015) |
| 6. Modified Western Ontario and McMaster University Osteoarthritis Index Scale | Shen Z. et al (2019) |
| 7. Tegner Activity Score | Hongshi Huang et al(2016) |
| 8. Forgotten Joint Score(FJS) | Shiqi Cao et al (2017) |
| 9. University of California at Los Angeles Activity Score for Arthroplasty and Arthroscopy | Shiqi Cao et al (2017) |
| 10. Anterior Cruciate ligament return to Sport after injury Scale(ACL-RSI) | Tian Wu Chen et al (2017) |
| 11. New Knee Society Scoring System(NKSS) | Liu et al (2015) |
| 12 .Knee Outcome Survey Activities of Daily Living Scale(KOS-ADLS) | Zhen-yu Jia et al (2016) |
| 13. Activity and Participation Questionnaire(APQ) | Cheng Chen et al (2020) |
| 14. Western Ontario Meniscal Evaluation Tool(WOMET) | Tong W.W et al(2016) |
| 15. Hospital for special surgery total knee replacement expectations survey(HSS-TKRES) | Tian Rui Rui (2016) |
| 16. The Osteoarthritis of Knee and Hip Quality of Life(OAKHQOL) | W.Wang et al (2016) |
| 17. Lower Extremity Function Scale(LEFS) | Ling Xu et al (2020) |
| 18. Intermittent and constant osteoarthritis pain(ICOAP) | C. Zhang et al (2017) |
| 19. International Physical activity questionnaire(IPAQ) | Lan Ping Wen et al (2013) |
| 20. Extended Nursing demand Scale | Wu Su Qin et al (2016) |
| 21. Frenchay Activity assessment scale | Zhang Ting Jiu et al (2014) |
| 22. Immobilization Comfort Questionnaire | Zhang li et al (2018) |
| 23. Japanese Knee Osteoarthritis measure | Wu Yan et al (2014) |
| 24. Self-efficacy for rehabilitation Outcome scale | Wang Hai Yan et al(2014) |
| 25. Musculoskeletal quality of Health | Pu Ying (2019) |
| 26. Adherence to home-based rehabilitation | Zhao Gai Yun (2019) |
| 27. Knee Self Efficacy Scale | Zhao Hui (2015) |
| 28. Knee Osteoarthritis Questionnaire | Huan Song Wei et al (2013) |
| 29. Knee Osteoarthritis Traditional Medicine syndrome PRO scale | Huang Song Min (2017) |
| 30. Tampa Scale for Kinesiophobia-11 (TSK-11) | Libai Cai et al (2019) |
| 31. Groningen Orthopedic Social Support Scale | SHENG Xiao-Juan et al(2019) |
| **Hip Instruments (n=10)** |  |
| 1.Oxford Hip Score(OHS) | Xia Zhen-lan et al (2012) |
| 2. Hip disability and Osteoarthritis Outcome(HOOS) | X. Wei et al (2012) |
| 3. Copenhagen Hip and Groin Outcome Score (HAGOS) | Shi Qi Cao et al (2018) |
| 4. International Hip Outcome Tool (SC-iHOT-33) | D.H. Li et al (2016) |
| 5. Osteoarthritis of Knee and Hip Quality of Life (OAKHQOL) | W. Wang et al. (2016) |
| 6. Hospital for Special Surgery Hip Replacement Expectations Survey (HSS-THRES) | Chen Wang et al (2018) |
| 7. Groningen Orthopedic Social Support Scale | SHENG Xiao-Juan et al(2019) |
| 8. Discharge Scoring Scale (PTHRDSS) | WangXiaoYan (2016) |
| 9. Abbreviated eight item measure of Penn State Worry Questionnaire (PSWQ-A) | Liu Yanjin et al (2015 ) |
| 10. Questionnaire on the perceptions and functions of patients about Total Hip Arthroplasty | Tang Hong Yuan et al (2010) |

See additional file 1 for references
