# Supplementary material for: Exploring the impact of Patient Reported Outcome Measures (PROMs) among orthopaedic surgeons in mainland China: systematic review and survey-based study on hip and knee instruments
Source: BMC Musculoskelet Disord. 2021 Jun 21;22:566. doi: 10.1186/s12891-021-04459-3 (PMC8218500; doi:10.1186/s12891-021-04459-3)
Supplement: Supplementary file 3 — Additional file 3. Chinese knee and hip score references. List of references for all the scores included in table. [file 12891_2021_4459_MOESM3_ESM.docx]

**References of Instruments for Table 1**

**Knee Instruments**

1. Jia, Zy., Zhang, C., Zou, Y. et al. Translation and validation of the Simplified Chinese version of International Knee Documentation Committee Subjective Knee Form. Arch Orthop Trauma Surg 138, 1433–1441 (2018). <https://doi.org/10.1007/s00402-018-2973-2>

2. Wang. Jian Sheng. Validation and reliability of the Chinese Version of the Knee Injury and Osteoarthritis Outcome Score for patients with injury. Master’s Dissertation. Shantou University, 2011/04.

3.Wang W, Liu L, Chang X, Jia ZY, Zhao JZ, Xu WD. Cross-cultural translation of the Lysholm knee score in Chinese and its validation in patients with anterior cruciate ligament injury. BMC Musculoskelet Disord. 2016 Oct 19;17(1):436. doi: 10.1186/s12891-016-1283-5. PMID: 27756266; PMCID: PMC5069932.

4. Lin K, Bao L, Wang J, Fujita K, Makimoto K, Liao X. Validation of the Chinese (Mandarin) Version of the Oxford Knee Score in Patients with Knee Osteoarthritis. Clin Orthop Relat Res. 2017 Dec;475(12):2992-3004. doi: 10.1007/s11999-017-5495-2. Epub 2017 Sep 7. PMID: 28884273; PMCID: PMC5670067.

5. Symonds T, Hughes B, Liao S, Ang Q, Bellamy N. Validation of the Chinese Western Ontario and McMaster Universities Osteoarthritis Index in Patients From Mainland China With Osteoarthritis of the Knee. Arthritis Care Res (Hoboken). 2015 Nov;67(11):1553-60. doi: 10.1002/acr.22631. PMID: 26018634.

6. Shen ZD, Yu HM, Wang JT, Shi GY, Sun Y. [Modified Western Ontario and McMaster University Osteoarthritis Index Scale used in patients with knee osteoarthritis]. Zhonghua Yi Xue Za Zhi. 2019 Feb 19;99(7):537-541. Chinese. doi: 10.3760/cma.j.issn.0376-2491.2019.07.012. PMID: 30786353.

7. Huang H, Zhang D, Jiang Y, Yang J, Feng T, Gong X, Wang J, Ao Y. Translation, Validation and Cross-Cultural Adaptation of a Simplified-Chinese Version of the Tegner Activity Score in Chinese Patients with Anterior Cruciate Ligament Injury. PLoS One. 2016 May 17;11(5):e0155463. doi: 10.1371/journal.pone.0155463. PMID: 27186880; PMCID: PMC4871333.

8. Cao S, Liu N, Han W, Zi Y, Peng F, Li L, Fu Q, Chen Y, Zheng W, Qian Q. Simplified Chinese version of the Forgotten Joint Score (FJS) for patients who underwent joint arthroplasty: cross-cultural adaptation and validation. J Orthop Surg Res. 2017 Jan 14;12(1):6. doi: 10.1186/s13018-016-0508-5. PMID: 28088227; PMCID: PMC5237477.

9. Cao S, Liu N, Li L, Lv H, Chen Y, Qian Q. Simplified Chinese Version of University of California at Los Angeles Activity Score for Arthroplasty and Arthroscopy: Cross-Cultural Adaptation and Validation. J Arthroplasty. 2017 Sep;32(9):2706-2711. doi: 10.1016/j.arth.2017.03.057. Epub 2017 Apr 13. PMID: 28483213.

10. Chen T, Zhang P, Li Y, Webster K, Zhang J, Yao W, Yin Y, Ai C, Chen S. Translation, cultural adaptation and validation of simplified Chinese version of the anterior cruciate ligament return to sport after injury (ACL-RSI) scale. PLoS One. 2017 Aug 17;12(8):e0183095. doi: 10.1371/journal.pone.0183095. PMID: 28817645; PMCID: PMC5560729.

11. Liu D, He X, Zheng W, Zhang Y, Li D, Wang W, Li J, Xu W. Translation and validation of the simplified Chinese new Knee Society Scoring System. BMC Musculoskelet Disord. 2015 Dec 21;16:391. doi: 10.1186/s12891-015-0854-1. PMID: 26691170; PMCID: PMC4687130.

12. Jia ZY, Wang W, Nian XW, Zhang XX, Huang ZP, Cui J, Xu WD. Cross-cultural Adaptation and Validation of the Simplified Chinese Version of the Knee Outcome Survey Activities of Daily Living Scale. Arthroscopy. 2016 Oct;32(10):2009-2016. doi: 10.1016/j.arthro.2016.01.068. Epub 2016 Apr 28. PMID: 27132769.

13.Chen C, Wang W, Wu H, Gao A, Qiu Y, Weng W, Price A. Cross-cultural translation and validation of the Chinese Oxford Knee Score and the Activity and Participation Questionnaire. J Orthop Surg (Hong Kong). 2020 Jan-Apr;28(2):2309499020910668. doi: 10.1177/2309499020910668. PMID: 32301381.

14. Tong WW, Wang W, Xu WD. Development of a Chinese version of the Western Ontario Meniscal Evaluation Tool: cross-cultural adaptation and psychometric evaluation. J Orthop Surg Res. 2016 Aug 15;11(1):90. doi: 10.1186/s13018-016-0424-8. PMID: 27523717; PMCID: PMC4983781.

15. Tian Rui Rui; Patient outcome expectations of knee arthroplasty and the association with post-operative function and satisfaction. Master’s Dissertation. Tianjin Medical University. 2016/05.

16. W. Wang, C.R. He, W. Zheng, J. Li, W.D. Xu. Development of a valid simplified Chinese version of the Osteoarthritis of Knee and Hip Quality of Life (OAKHQOL) in patients with knee or hip osteoarthritis. J Eval Clin Pract, 4 (2015)

17. Xu, L., Wang, C., Zhang, C. et al. Cross-cultural adaption and validation of simplified Chinese version of the lower extremity function scale in patients with knee osteoarthritis.Clin Rheumatol 39, 3041–3048 (2020). <https://doi.org/10.1007/s10067-020-05077-5>

18. C. Zhang, D.H. Liu, Y.L. Qu, Z.Y. Jia, W. Wang, J. Li, W.D. Xu.Transcultural adaptation and validation of the Chinese version of the intermittent and constant osteoarthritis pain (ICOAP) measure in patients with knee osteoarthritis. Osteoarthritis and Cartilage,Volume 25, Issue 4,2017,Pages 506-512,

ISSN 1063-4584,https://doi.org/10.1016/j.joca.2016.11.012.

19. Lan, P. and Shen, B. and Yang, J. The reliability and validity of international physical activity questionnaire for assessing the activity level of total knee replacement patients. Chinese Journal of Rehabilitation Medicine; 2013, 28(8): 743-746

20. Wu Su-Qin, Zhang Xi-Shun, Chen Yan-Qin, Feng Xiu-We. Reliability and Validity of Extending Nursing Demand Scale of Total Knee Replacement Patients . Journal of Nursing(China) . Vol.23 No.16

21. Tingjiu Zhang , Dong Zeng Fanwei，et al ; Reliability and Validity Analysis Activity Assessment in Elderly Patients after Total Knee Arthroplasty. . Sichuan Medical Journal，2014，Vol. 35，No. 12

22. Zhang Li, He Li, Meng Shanshan, Wang Yali. Analysis of the reliability and validity of the Chinese version of Immobilization Comfort Questionnaire among hospitalized total knee arthroplasty post-operative patients . Chin J Mod Nurs，February 26，2018，Vol.24，No.6

23. Xu Shou Yu, Yao Xin Miao, Wu Yan, et al. Reliability of the Chinese version of Japanese knee osteoarthritis measure . Chinese Journal of Rehabilitation Medicine, 2014, 29(8): 723—725

24. Wang Haiyan, Xu Yanling, Hu Sanlian, Zhou Ling, Qian Yan, Qian Huijuan. Evaluation of the reliability and validity of Chinese version self-efficacy for rehabilitation outcome scale; Chin J Mod Nurs, January 26, 2014, vol.20, No. 3

25. Pu Ying. Chinese Version of Musculoskeletal Health Questionnaire and Preliminary Application in Patients Undergoing Total Knee Arthroplasty. Master’s Dissertation. Hainan Medical College. 2019/05

26. Zhao Gaiyun, Qian Huijuan, Wang Fanfan, Xu Yanling ; Development and validation of the home-based rehabilitation adherence scale for patients with total knee arthroplasty .J ournal of Nursing Science Jun.2019 Vol.34 No.12

27. Zhao Hui . Reliability and validity of the Chinese version of the Knee Self-Efficacy Scale . Journal of Nursing Science Nov.2015 Vol.30 No.22

28. Huan Song-wei1, Tam Man-seng, CHEANG Lek-hang, Xia Ji-sheng, Wu Hao, You Tian, Zha Zhen-gang. Design and evaluation of the questionnaire about knee osteoarthritis; Chinese Journal of Tissue Engineering Research May 28, 2013 Vol.17, No.22

29. Huang Song Min. Knee Osteoarthritis Traditional Medicine syndrome PRO scale reliability, validity and responsiveness evaluation. Master’s Dissertation. Beijing University of Chinese Medicine, 2017/05.

30. Cai L, Liu Y, Woby SR, Genoosha N, Cui M, Guo L. Cross-Cultural Adaptation, Reliability, and Validity of the Chinese Version of the Tampa Scale for Kinesiophobia-11 Among Patients Who Have Undergone Total Knee Arthroplasty. J Arthroplasty. 2019 Jun;34(6):1116-1121. doi: 10.1016/j.arth.2019.01.076. Epub 2019 Feb 6. PMID: 30853160.

31. Sheng Xiao-Juan , Chen Wen-Yue , Fu Qiao-Mei . Translation of Groningen Orthopedic Social Support Scale into Chinese and Its Reliability and Validity in Patients after Joint Replacement of Hip and Knee. Journal of Nursing(China). Vol.26 No.14

**Hip Instruments**

1. Xia Zhen-lan, He Bing, Fan Mei-xia, Liu Xue-qin . Reliability and Validity of Chinese Version of Oxford Hip Score . Journal of Nursing(China) . March，2012. Vol.19 No.3A

2. Wei X, Wang Z, Yang C, Wu B, Liu X, Yi H, Chen Z, Wang F, Bai Y, Li J, Zhu X, Li M. Development of a simplified Chinese version of the Hip Disability and Osteoarthritis Outcome Score (HOOS): cross-cultural adaptation and psychometric evaluation. Osteoarthritis Cartilage. 2012 Dec;20(12):1563-7. doi: 10.1016/j.joca.2012.08.018. Epub 2012 Aug 31. PMID: 22944526.

3. Cao S, Cao J, Li S, Wang W, Qian Q, Ding Y. Cross-cultural adaptation and validation of the Simplified Chinese version of Copenhagen Hip and Groin Outcome Score (HAGOS) for total hip arthroplasty. J Orthop Surg Res. 2018 Nov 6;13(1):278. doi: 10.1186/s13018-018-0971-2. PMID: 30400966; PMCID: PMC6219004.

4. Li DH, Wang W, Li X, Gao YL, Liu DH, Liu DL, Xu WD. Development of a valid Simplified Chinese version of the International Hip Outcome Tool (SC-iHOT-33) in young patients having total hip arthroplasty. Osteoarthritis Cartilage. 2017 Jan;25(1):94-98. doi: 10.1016/j.joca.2016.08.013. Epub 2016 Sep 9. PMID: 27621215.

5. Wang, W.,  He, C.‐R.,  Zheng, W.,  Li, J., and  Xu, W.‐D. (2016)  Development of a valid simplified Chinese version of the Osteoarthritis of Knee and Hip Quality of Life (OAKHQOL) in patients with knee or hip osteoarthritis. Journal of Evaluation in Clinical Practice,  22:  53– 61. doi: [10.1111/jep.12431](https://doi.org/10.1111/jep.12431).

6. Wang C, Zhang C, Liu DL, Tong WW, He CR, Huang X, Xu WD. Simplified Chinese version of hip and knee replacement expectations surveys in patients with osteoarthritis and ankylosing spondylitis: cross-cultural adaptation, validation and reliability. BMC Musculoskelet Disord. 2018 Jul 21;19(1):247. doi: 10.1186/s12891-018-2129-0. PMID: 30031384; PMCID: PMC6054857.

7. Sheng Xiao-Juan , Chen Wen-Yue , Fu Qiao-Mei . Translation of Groningen Orthopedic Social Support Scale into Chinese and Its Reliability and Validity in Patients after Joint Replacement of Hip and Knee. Journal of Nursing(China). Vol.26 No.14

8. WangXiaoYan .Localization and Clinical application of the Post Total Hip Replacement . Master’s thesis. 2016/11

9. Liu Yanjin, Gao Huan Huan, Zhao Hui. Reliability and validity of Chinese Version of an abbreviated eight item measure of Penn State Worry Questionnaire in patients after total hip replacement. Chin J Prac Nurs，September 21 2015，Vol.31，No.27

10. Tang Hong-Yuan, Yang Ming-Yu, Zhang Li-Ming. Development and Evaluation of questionnaire on the perceptions and function of patients about total hip arthroplasty. Chin J Mod Nurs, November 2010, Vol 16, No.33.
